# On the Exhibition of Ergot of Rye in Lingering Labor, and the Conditions for Its Safe Employment

**Published:** 1846-06

**Authors:** 


6. *On the Exhibition of Ergot of Rye in lingering Labor, and the Conditions for its safe employment.*—[S. Hall Davis, M.D., Lecturer on Midwifery, and Physician to the Maternity Charity, considers that the following conditions should be present in order to render the exhibition of the medicine a safe proceeding:]

1. The soft parts should be lax, and free from heat.

2. With rare exceptions, the orifice of the uterus should be nearly fully dilated, and always dilateable.

3. The pelvic space should present the average dimensions.

4. In head presentations only; in breech and footling cases it is objectionable, on account of the very gradual descent of the presenting parts required for the subsequent passage of the head without risk to the cord. In transverse presentations it is obviously improper.

5. The head should be in an average good position, and not impacted.

6. The inertia of the womb should not have its source in plethora.

7. The absence of any source of irritation in any other organ capable of disturbing the parturient function by reflex action should first be ascertained, as of fæcal accumulation, urine in the bladder, or crude ingesta, which are to be met by their obvious indications of treatment.

8. The uterine inertia should be ascertained not to depend upon disturbance of the nervous functions, from loss of rest, or from depressing emotions.

9. There should not be present any cause of distension of the womb, beyond its power of acting to advantage, as by excessive quantity of liquor amnii or twins.

10. It would not be indicated when the inertia arises from constitutional weakness.

11. It is rarely advisable in primiparæ; much time and slower parturient action being required in these cases.

In short, this remedy being indicated on account of inertia of the womb, arising from the effect of previous distensions, there should be for its safe exhibition, a natural presentation, a wide pelvis, soft parts relaxed, nothing wanting, in a word, but efficient action of the uterus to finish the labor.—*Ranking's Abstract*, Part II.

